# Gibberellic acid in *Citrus* spp. flowering and fruiting: A systematic review

**DOI:** 10.1371/journal.pone.0223147

**Published:** 2019-09-26

**Authors:** Alfonso Garmendia, Roberto Beltrán, Carlos Zornoza, Francisco J. García-Breijo, José Reig, Hugo Merle

**Affiliations:** 1 Instituto Agroforestal Mediterráneo, Universitat Politècnica de València, Valencia, Spain; 2 Departamento de Ecosistemas Agroforestales, Universitat Politècnica de València, Valencia, Spain; 3 S.A. Explotaciones Agrícolas Serrano (SAEAS), Picassent, Valencia, Spain; 4 Instituto Cavanilles de Biodiversidad y Biología Evolutiva, Jardín Botánico Universitat de València, Valencia, Spain; United States Department of Agriculture, UNITED STATES

## Abstract

**Background:**

In *Citrus* spp., gibberellic acid (GA) has been proposed to improve different processes related to crop cycle and yield. Accordingly, many studies have been published about how GA affects flowering and fruiting. Nevertheless, some such evidence is contradictory and the use of GA applications by farmers are still confusing and lack the expected results.

**Purpose:**

This review aims to collate, present, analyze and synthesize the most relevant empirical evidence to answer the following questions: (i) how does gibberellic acid act on flowering and fruiting of citrus trees?; (ii) why is all this knowledge sometimes not correctly used by farmers to solve yield problems relating to flowering and fruit set?

**Methods:**

An extensive literature search to obtain a large number of records about the topic was done. Searches were done in five databases: WoS, Scopus, Google Academics, PubMed and Scielo. The search string used was "Gibberellic acid" AND "Citrus". Records were classified into 11 groups according to the development process they referred to and initial data extraction was done. Records related with flowering and fruit set were drawn, and full texts were screened. Fifty-eight full text records were selected for the final data extraction.

**Results:**

Selected studies were published from 1959 to 2017 and were published mainly in Spain, USA, Brazil and Japan. Twelve species were studied, and *Citrus sinensis*, *C*. *reticulata* and *C*. *unshiu* were the principal ones. Most publications with pre-flowering treatments agreed that GA decreases flowering, while only 3 out of 18 did not observe any effect. In most of these studies, the effect on fruit set and yield was not evaluated. Studies with treatments at full bloom or some weeks later mostly reported increased fruit set. However, these increases did not imply higher yields. The results on yield were highly erratic as we found increases, decreases, no effects or variable effects.

**Conclusions:**

Despite some limitations, the action of GA related to cell division and growth, stimulating the sink ability of the organ and discouraging its abscission, has been clearly established through reviewed studies. GA applications before flowering counteract the floral induction caused by stress reducing flowering. However, on adult trees under field conditions, reducing flowering by applying GA would be difficult because it would be necessary to previously estimate the natural floral induction of trees. During flowering and fruit set, many problems may arise that limit production. Only when the problem is lack of fruit set stimulus can GA applications improve yields. However, much evidence suggests that the main factor-limiting yield would be carbohydrate availability rather than GA levels. GA applications increased fruit set (often transiently), but this increase did not mean improved yields.

## Introduction

Gibberellic acid (GA) is a well-known plant hormone [1]. In *Citrus* spp. and other crops, GA has been proposed to improve different crop cycle- and yield-related processes [2–8]. Certain citrus varieties can have different yield-related problems [9,10]. Farmers’ goal is to produce high good-quality yields every year. To achieve this objective in fruit trees, it is essential to adjust flowering, fruit set and yield (fruit number and size) to trees’ annual capacity [10]. If these values go over the tree-holding capacity, high yields, but small fruit and tree weakening, will be obtained. However, if these values go below the tree-holding capacity, low yields will result [9,10].

Normally trees do this adjustment themselves naturally as they produce many more flowers than they need and then reduce the number to balance it with their capacity [11]. However, sometimes trees can fail this adjustment, in which cases farmers can mediate to obtain a better result. Farmers have to deal with different situations depending on variety behavior (Table 1).

**Table 1:** Problem scheme according to variety behavior.

Starting point	from flowering to harvest			End-point
Variety	Behavior	Problem	Solution	Farmers’ goal
Productive and non alternate. e.g. Navelina	Adequately adjusting reproductive growth to tree capacity	Variety without problems	Farmers do not need to act in the process	High yield Good quality (size) Stable over years
Always unproductive e.g. Orri	Produces few or many flowers bud with low fruit set	Small yield	Farmers must encourage reproductive growth	
Always excessively productive e.g. Fine Clementine	Excessive flowers and / or fruit set.	Many small fruits	Farmers must limit reproductive growth	
Alternate cv. to a greater or lesser extent, e.g. Nadorcott	Years with good yields: small fruits and exhausted trees Years with poor yields	Years with good yields and small size. Years with poor yields	Farmers must act to reduce excess	

non alternate var., variety with constant yield over the years; reproductive growth, production and development of reproductive organs (flowers); exhausted trees, trees with no carbohydrate reserves.

The most characteristic problems are: (i) excessive tree weakening due to excessive yields; it is worth noting the behavior of var. Murcott (*Citrus reticulata* Blanco), which may set a very heavy crop that causes the tree to die [9,12]; (ii) small fruit size caused by heavy flowering and crops, which sometimes need thinning, a common expensive practice in "on" years with small fruits [13–15]; (iii) constant low fruit set, which sometimes requires branch girdling, another expensive practice [16–18]; finally, one of the commonest problems (iv) is alternate bearing [5,19,20].

The majority of citrus varieties show alternate bearing to a greater or lesser extent [10,21,22]. Not only individual farmers, but also on a large territorial scale, "on" and “off” years are usual. In “on” years, high productions are obtained by most farmers and varieties, which will produce low quality (small fruits) and excess supply and, therefore, low prices [20]. Thus, probably “on” and “off” years are somewhat synchronized by climate conditions. Alternate bearing does not necessarily mean biannual on-off years [22] as there may be intermediate yields depending on the climate, the tree’s reserves and interactions via agronomic practices.

However, problem identification is just the first step; and then, how can GA applications help farmers deal with these circumstances? Hundreds of studies have been published about the effect of GA on flowering and fruiting in *Citrus* spp. Some such evidence is contradictory and the use of GA applications by farmers under field conditions is still confusing and does not always lead to the expected results. GA_3_ is regularly used in the production of citrus to improve yield, but several authors agree that the problem that limits the use of these plant hormones is their unpredictable performance [23,24].

Systematic reviews allow problems to be analyzed with highly variable results to help understand this variability. Traditional reviews may fail in selecting those studies that argue authors´ initial points of view, while systematic reviews are based on nonbiased data extraction from a subset of studies that fit the pre-established eligibility criteria. Systematic reviews aim to find a robust and sensible answer to a focused research question.

Therefore, this paper proposes the first systematic review on GA in *Citrus* spp. It aims to collate, present, analyze and synthesize the most relevant empirical evidence to answer the following questions: (i) how does GA acts on the flowering and fruiting of *Citrus* trees?; (ii) why is all this knowledge sometimes not correctly used by farmers to solve yield problems relating to flowering and fruit set? Although a brief review of other processes is carried out, this paper focuses on the effect of exogenous GA applications on citrus flowering and fruiting, and their relations with yield.

## Materials and methods

The Preferred Reporting Items for Systematic Reviews and Meta-Analyses (PRISMA) guidelines were followed [25]. This methodology summarizes the evidence available on a topic to convey its breadth and depth.

### Research question

The main research question for this review was: how does GA act on the flowering and fruiting of *Citrus* trees?

### Information sources and search strategy

First, a broad literature search to obtain lots of records on the topic was done. Searches were carried out in five databases: WoS, Scopus, Google Academics, PubMed and Scielo. The search string used in all databases was "Gibberellic acid" AND "Citrus" and, whenever possible, it was limited to the title, abstract and key words (Table 2). Searches were not limited to publish dates (all years), document type (all) or language (all).

**Table 2:** Electronic search strategy.

Database	Specific search string	Published	Doc type	Lang.	n =
WoS	THEME: (Gibberellic acid) AND (Citrus)	all years	all	Auto	463
Scopus	TITLE-ABS-KEY ("Gibberellic acid" AND "Citrus")	all years	all	all	158
Google Academics	allintitle: Gibberellic acid Citrus	all years	all	all	130
PubMed	(Gibberellic acid[Title/Abstract]) AND Citrus[Title/Abstract]	all years	all	all	13
Scielo	All: (Gibberellic acid) AND (Citrus)	all years	all	all	23

### Initial classification of records and first data collection

**Fig 1:**
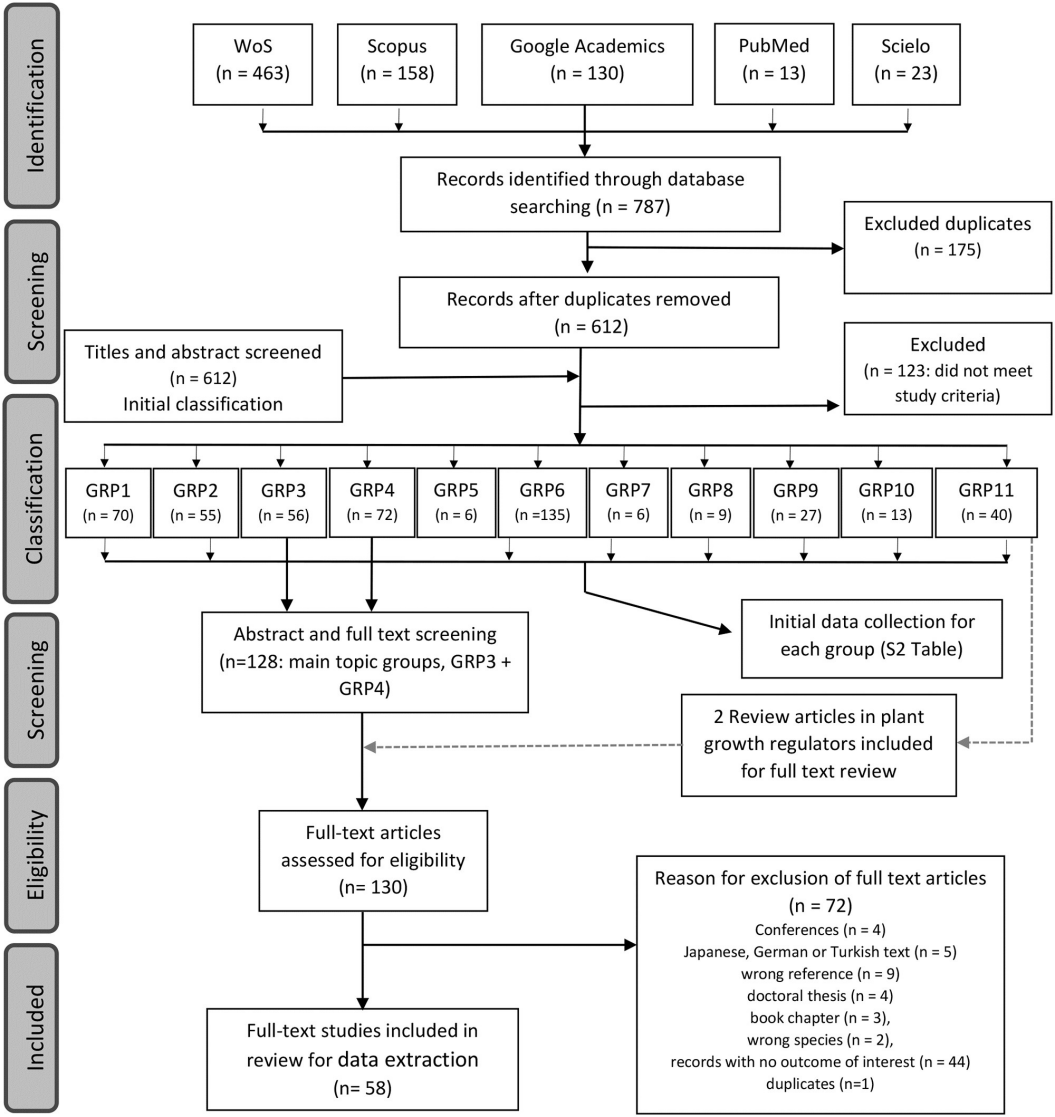
Overview of the article selection process. Papers were collected considering the search string in the databases.

From the identified records (n = 787), duplicates were removed (Fig 1). The remaining records (n = 612) were classified into 11 groups according to the development process they referred to (S1 Table). At this point, an initial data collection process was followed for each group to obtain an overview of GA action on not only on flowering and fruit set, but also on other development processes and species. To that end, abstracts were reviewed and the main results were charted (S2 Table).

### Eligibility criteria for a full text review

Group 3 (flowering records; n = 56), group 4 (fruit set and yield records; n = 72) and two review articles from group 11 were abstract- and full text-screened (n = 130) (Fig 1). Conferences (n = 4), in Japanese, German or Turkish (n = 5), wrong references (n = 9; mistakes in the title or journal data), doctoral theses (n = 4); book chapters (n = 3), wrong species (n = 2), records with no outcome of interest (n = 44; very low quality studies, remoteness of results from the main topic, etc.) and duplicates (n = 1) were excluded. Therefore, 58 records were selected for the final data extraction (Fig 1).

### Charting data

The data from the 58 records were included in a table (S3 Table) with the following items:

- Article identifiers: authors, year of publication, country, title.
- Study information: species, variety, phase in which GA was applied (pre-flowering; post-flowering; small fruit), study type (experiment or observation).
- Treatments: experiment scale (if the experiment was conducted on individual flowers, trees or rows of trees (field)), tree age, concentration (mg. l^-1^ of GA in the solution), years of experiments (number of years during which the experiments were conducted).
- Experiment size.
- Main result text.
- Main result tabulated: N (no effect), I (increases), D (decreases), O (not evaluated), V (variable effect).
- Conclusion text.
- Interest: ‘interest scale’ goes from 1 to 5, and reflects the closeness to the main topic; ‘reliability scale’ goes from 1 to 3, (low, medium, high) and reflects the quality and reproducibility of experiments.

### Collating, summarizing, and reporting the results

A descriptive numerical summary of the characteristics of the included studies was prepared.

Tables and graphs were created to reflect the overall number of studies included, study designs and settings, publication years, reported outcomes, and the countries where studies were conducted. All the statistical analyses were done using R [26] and RStudio [27]. For the graphics, some additional packages were used: ggplot2 [28], alluvial [29], cowplot [30], RColorBrewer [31] and waffle [32].

## Results

### Search in databases and selecting papers

In all, 787 articles were retrieved from five databases. WoS contributed with most papers for this review, 59% of the total (Fig 1). The Scopus, Google Academics, PubMed and Scielo databases represented, respectively, 20%, 16%, 2%, and 3% of the papers found. The databases with a broader search spectrum, such as WoS, Scopus and Google Academic, retrieved the most records. Other databases could have been used, but adding more databases only increases duplicates.

### Initial classification and data extraction

**Fig 2:**
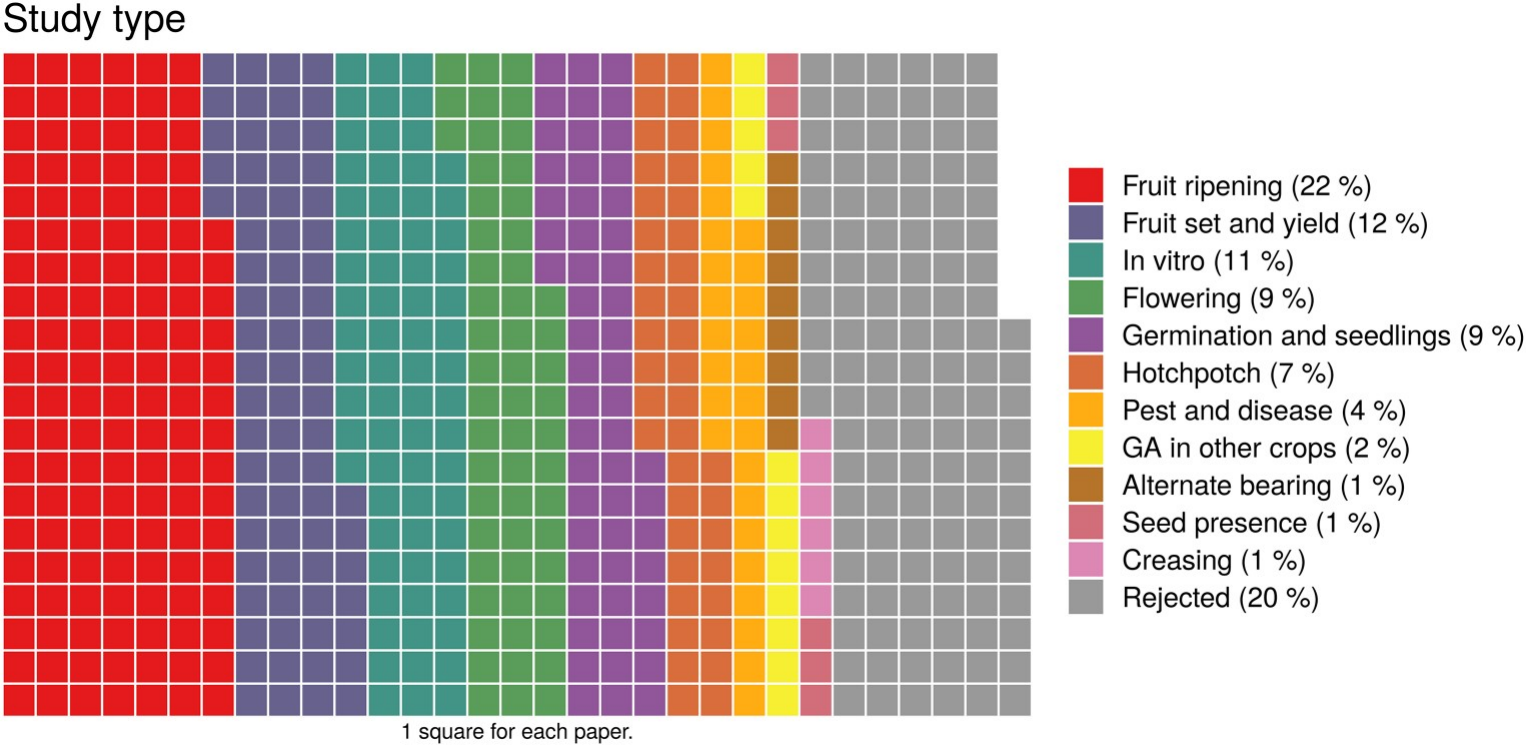
Classification of the 612 records in 11 groups according to the crop cycle process that GA affected.

Records were classified into 11 groups according to their content (S1 Table). Any records that clearly did not relate to the topic were rejected (n = 123; 20%) (Fig 2). The main group of records were about ‘fruit ripening’ (22%), including pre- and post-harvest GA treatments to prolong the storage quality of citrus fruits (Fig 2). Other important groups were ‘*in vitro* callus growth and embryogenesis’ with 11%, ‘germination and seedlings’ with 9%, ‘flowering’ with 9%, and ‘fruit set and yield’ with 12% (Fig 2).

To obtain an initial broad view, the main results of each group were tabulated (S2 Table). The ‘Pest and disease’ group was not examined given its remoteness from the topic. The ‘Flowering’ and ‘Fruit set and yield’ groups were not examined at this point as these records were moved to the next phase to continue the processes of screening, eligibility and data extraction. The results (S2 Table) of this initial phase were summarized (Table 3). It is necessary to clarify that not all the information from each group was studied in-depth, although a new and specific systematic review would be needed for each group or process. Only the information from the original string "Gibberellic acid" AND "Citrus" was reviewed to acquire the initial data and a broad view.

**Table 3:** Summary of the initial data extraction per process. Effect of GA on other *Citrus* development processes. Flowering, Fruit set and yield, and pest and disease groups are not included.

*In vitro callus growth*	Many growth regulators have been used in different phases, from callus growth to plant regeneration [33–39]. The effect of GA depends on the phases and the presence of other growth regulators. Most culture media contain GA at different concentrations, which seems more important toward final stages rather than in initial ones [34]. While the induction of embryogenesis from calli required GA in many casesd [36], in some others, adding inhibitors of GA_3_ to the medium enhanced embryogenesis [37].
*Germination and seedlings*	Most studies clearly showed how gibberellins enhanced seedling growth by increasing leaf number, stem length, internode length, root weight, dry weight, and carbon supply in shoots [2,3,40–43]. Many of these studies were also carried out with gibberellin-biosynthesis inhibitors, mainly Paclobutrazol (PBZ). In all these cases, GA_3_ reversed the action of PBZ [40,42]. The germination percentages of seeds were significantly increased by GA_3_ treatments [2,44], but not always. Some studies found no significant effect of GA treatments on final germination percentages (e.g. in grapefruit) [45,46].
*Seed presence*	GA treatments alone or combined with copper have been tested to lower the number of seeds per fruit with limited effectiveness (reduction around 30%) [47–49]. The pollination/fecundation process enhances GA in naturally pollinated Murcott and Moncada ovaries compared to unpollinated Murcott and Moncalina [7]. Active gibberellin GA_1_ levels in ovaries seem to be related with the presence of fertilized ovules and, therefore, with the ability to produce seeds [7]. Fruit set was much higher in self-pollinated or non-pollinated flowers with GA spray than in flowers with no GA spray [50]. It seems that fruit set depends strongly on the GA_1_ level achieved by ovaries [7].
*Fruit ripening*	Countless evidence shows that (GA_3_) applications delay *Citrus* fruit ripening [8,51–60]. GA_3_ treatments delayed senescence, softened rind, changed essential oil and the rind color rate, and reduced the rind crease severity [52,53]. In some cases, a greener rind color was associated with GA treatments [53,58]. GA_3_ increased the juice yield of processed oranges, but the results were inconsistent [57]. The data suggest that GA efficacy was not dependent on any application method, but on spray volume and GA dose [59]. GA_3_ delayed fruit coloration and rind softening, while 2,4-D (2,4-dichlorophenoxy acetic acid) significantly reduced fruit drop [56].
*Creasing*	Treatments likes CaCl_2_, Ca(NO_3_)_2_, Zn, Zn + NAA (naphthaleneacetic acid), NAA, NAA + GA_3_ and only GA_3_ have been tested to control creasing [6,61–63]. CaCl_2_ treatments (0.33%) cause unacceptable fruit drop and leaf damage [62]. The best results were obtained with two sequential sprays: the first with NAA in May and the second with GA_3_ in August which reduced the incidence of creasing from 36% to only 3% of the fruits [6]. The early NAA spray (May) thinned 14% of the fruitlets and increased the size of the remaining fruit [6]. Gibberellic acid by itself does not seem to be able to completely control creasing [61]. In addition, late applications can affect rind quality inducing regreening of fruit [62].
*Alternate bearing*	The attempts to control alternate cropping used GA to reduce flowering on previous-low-fruit-load trees and PBZ to promote flowering on previous-heavy-fruit-load trees, however the effects were not enough to break the alternate bearing dynamic [19]. The effectiveness of PBZ in promoting flowering in *Citrus* depends on the previous fruit load [20]. Medium-to-low fruit-load trees treated with PBZ significantly increased flowering, while heavy fruit load trees receiving the same amount of PBZ scarcely flowered [20]. GA at 25 ppm applied twice reduced flowering, but by the time fruit were mature, trees had fully compensated for this early reduction in fruit numbers [19]. Therefore, GA and PBZ did reduce or increase flowering but not enough to control alternate bearing.
*GA effects in other crops*	Gibberellic acid acted similarly in other species as it did in citrus [64–66]. Fruit-set and growth in tomato depended on the action of gibberellins [64]. GA_3_ trunk-injected in avocado reduced both inflorescence number and fruit set/inflorescence [66]. In persimmon, the interaction between increased ABA and decreased IAA and GA-like substances in the fruitlet before fruitlet-drop induced its abscission [65].
Hotchpotch	Three review articles were found within the search strategy [67–69]. These articles were full text reread for the following steps. Several articles studied interactions with other plant hormones and source-sink relationships [70,71]. Inflorescence leaves of *Citrus sinensis* accumulated carbohydrate reserves at the beginning of the fruit set period. This effect was mimicked by exogenous GA_3_ applications in deflorated inflorescences [70]. The data indicated that there were antagonistic changes between ABA and GA20, because in both species ABA increased and GA20 decreased during water stress, re-hydration via either rainfall or irrigation reduced ABA but increased GA20 [71].

### Main topic data extraction and studies’ characteristics

**Fig 3:**
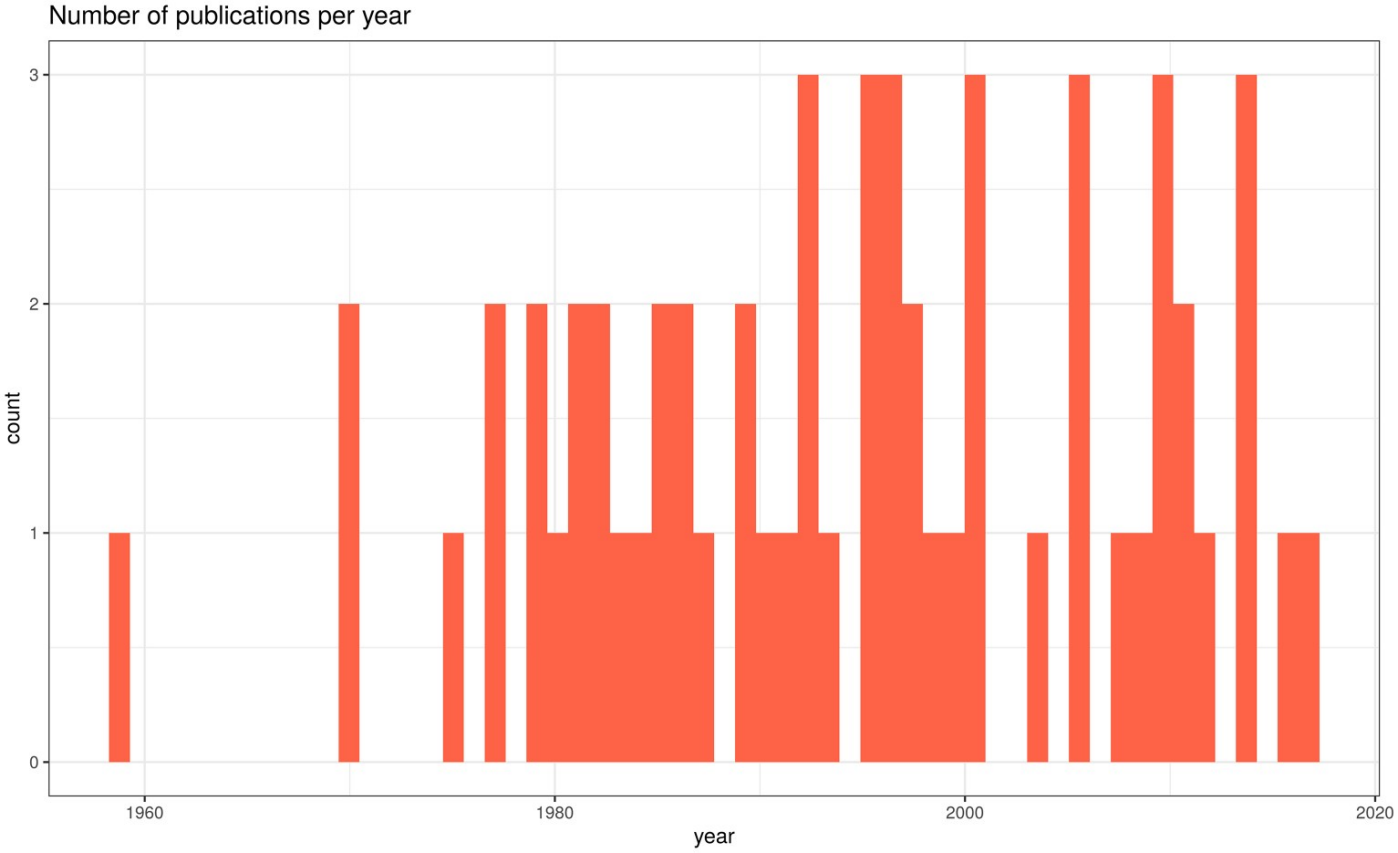
Number of publications per year, from 1959 to 2017.

**Fig 4:**
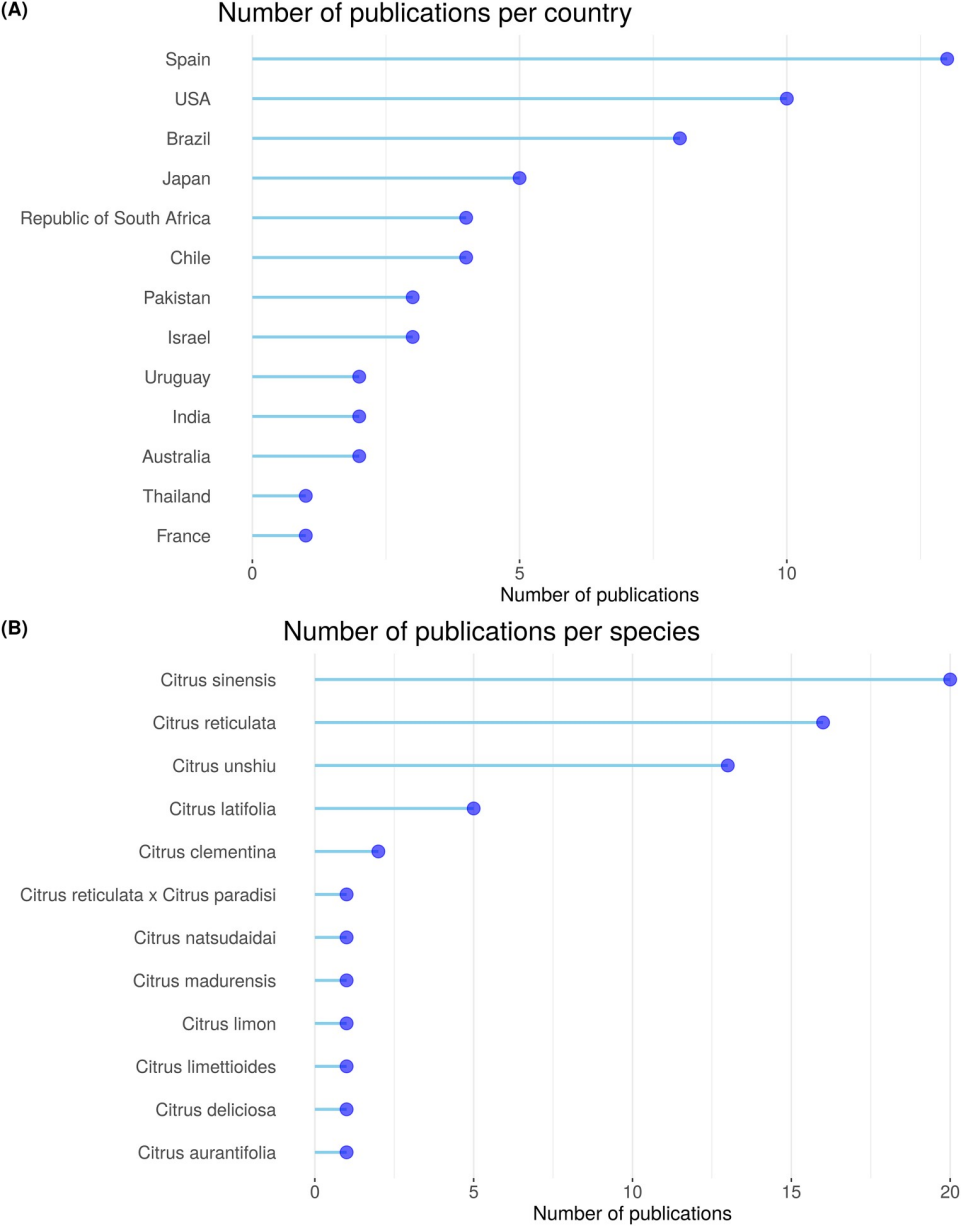
Number of publications per country (A) and per species (B).

After selection phase, only 21% or 130 papers out of a total of 612 were selected for the main topic review (groups 3 and 4 and 2 review articles). In the extraction phase, of the 130 previously selected papers, 45% were accepted and 55% were rejected by the exclusion criteria. Thus, 58 out of 130 papers related with the effect of GA on flowering, fruit set and yield were selected for data extraction (S3 Table). Studies were published from 1959 to 2017 (Fig 3) and the main countries of publication were Spain, USA, Brazil and Japan (Fig 4A). Twelve species were studied, being *Citrus sinensis* (L.) Osbeck, *C*. *reticulata* Blanco and *C*. *unshiu* Marc. the main ones (Fig 4B). Within the 12 species, 31 varieties were studied, being Nules (*C*. *reticulata*,) Navelate (*C*. *sinensis*) and Tahiti lime (*C*. *latifolia* Tan.) the most frequent ones (S3 Table). These varieties are parthenocarpic and seedless in the absence of cross-pollination.

**Fig 5:**
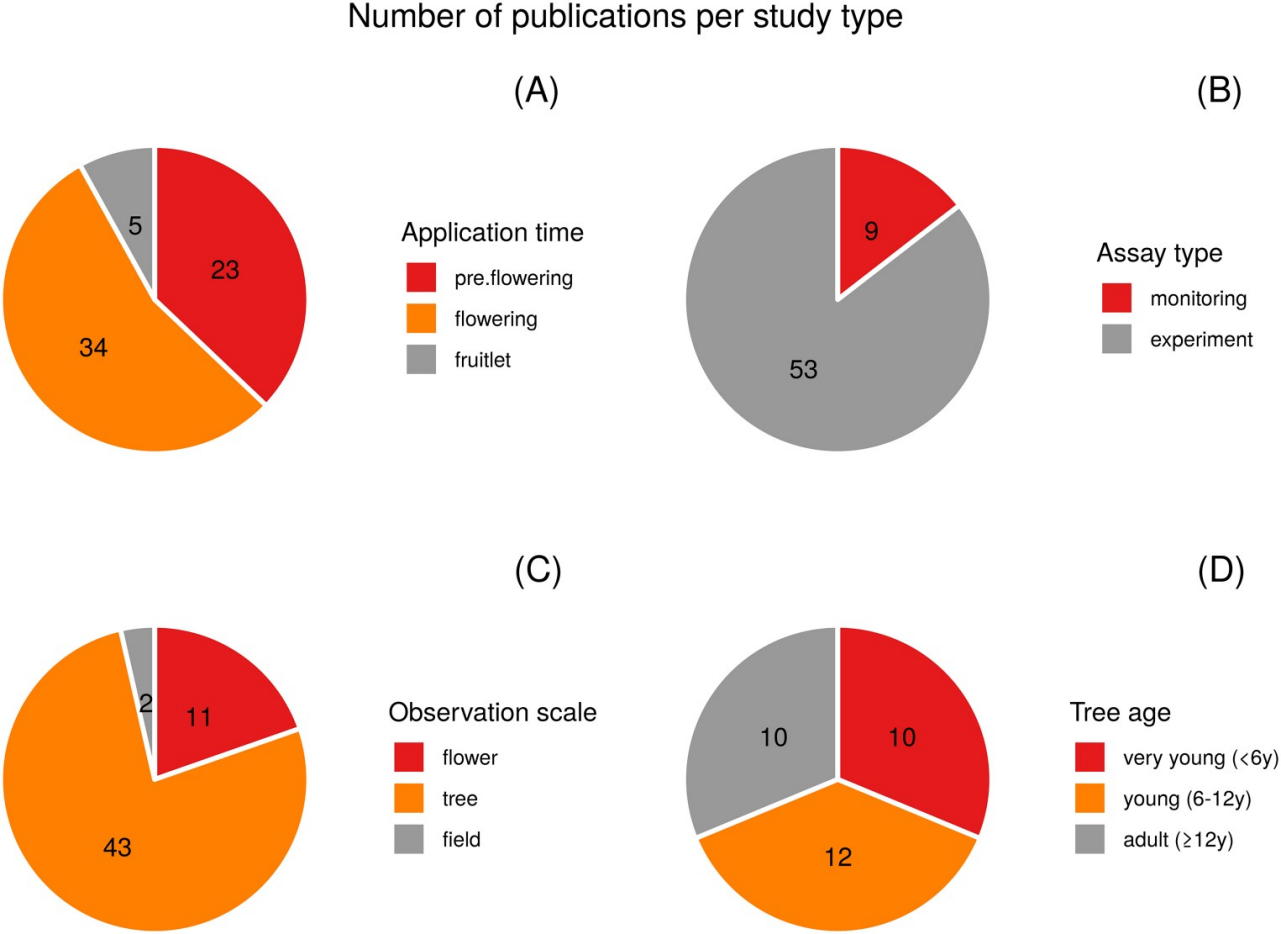
Characteristics of studies. Number of publications per application time (A), assay type (B), observation scale (C) and tree age (D). Note: the total number of publications does not necessarily have to be 58 as in some cases, e.g. tree age was not specified, and several observation scales were used in other cases.

**Fig 6:**
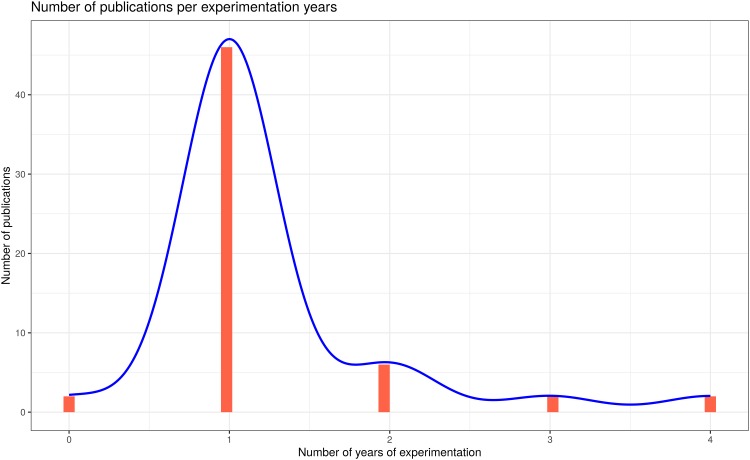
Number of publications per experimentation years for the 58 publications from 1959 to 2017 with indications of it.

GA applications were carried out before flowering (pre-flowering treatments; 37%), at full bloom or a few weeks later (flowering; 55%), or later during fruitlet growth (fruitlet; 8%) (Fig 5A). Most studies carried out experiments (85%), and some monitored GA contents and other substances throughout the flowering process (15%) (Fig 5B). The observation scale was mainly ‘tree’ (77%), but some experiments were carried out on inflorescence or individual flowers (20%), and very few had a bigger scale (3%) (Fig 5C). The studies that used full mature trees represented 31.5%, while most studies used trees under 12 years old (68.5%), of which almost half (31%) were under 6 years (Fig 5D). Most experiments were conducted for 1 year and very few were performed for 2 or 3 years (Fig 6).

**Fig 7:**
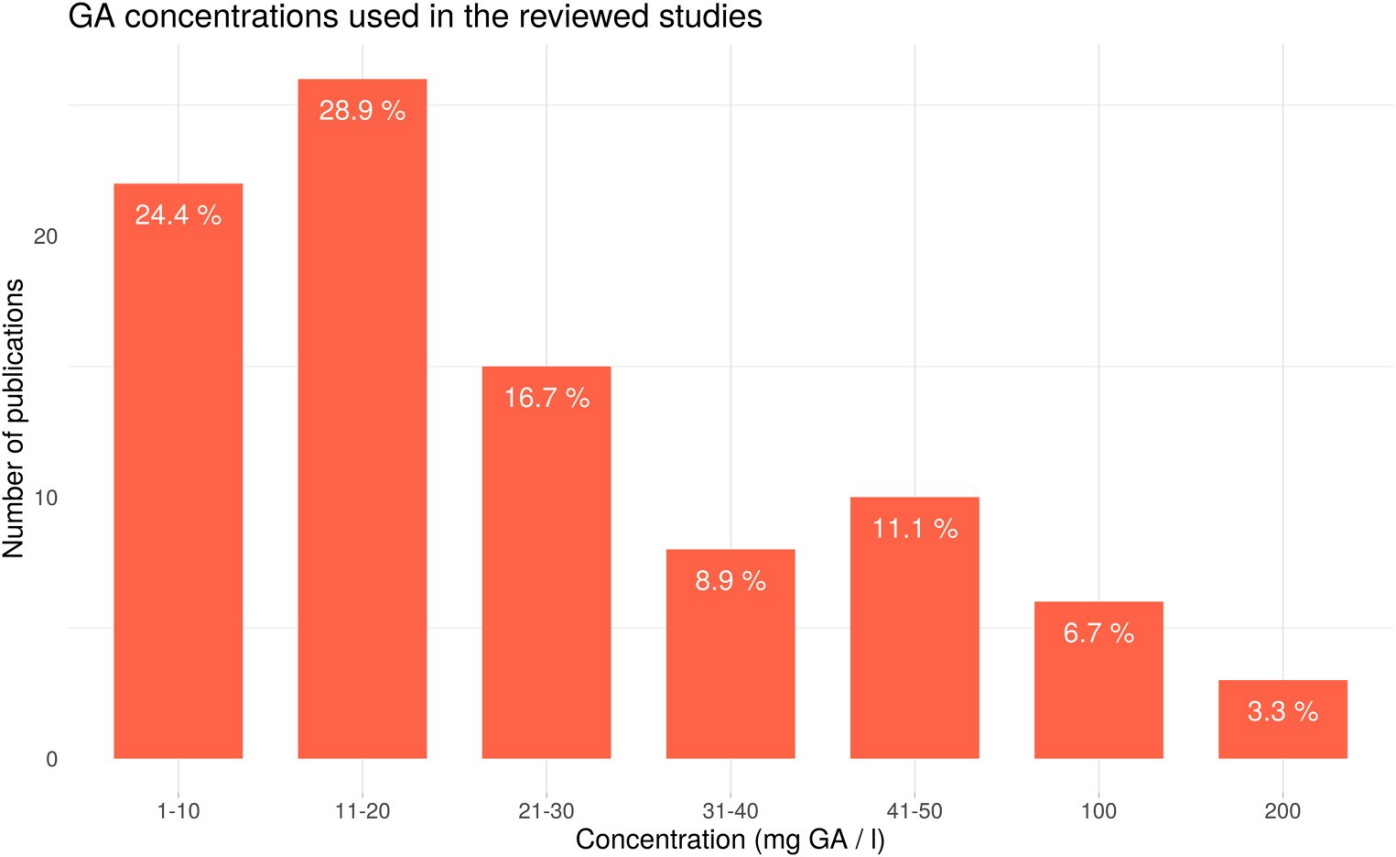
The GA concentrations used in the reviewed studies. For each concentration, the number of publications which used that concentration was noted. Percentages were calculated over the total amount of annotations.

The GA treatments expressed as mg.L^-1^ went from 10 to 200 (Fig 7). The most widely used concentrations were 11–20 (28.9%), 1–10 (24.4%), and 21–30 (16.7%) (Fig 7).

### The results of the studies

**Fig 8:**
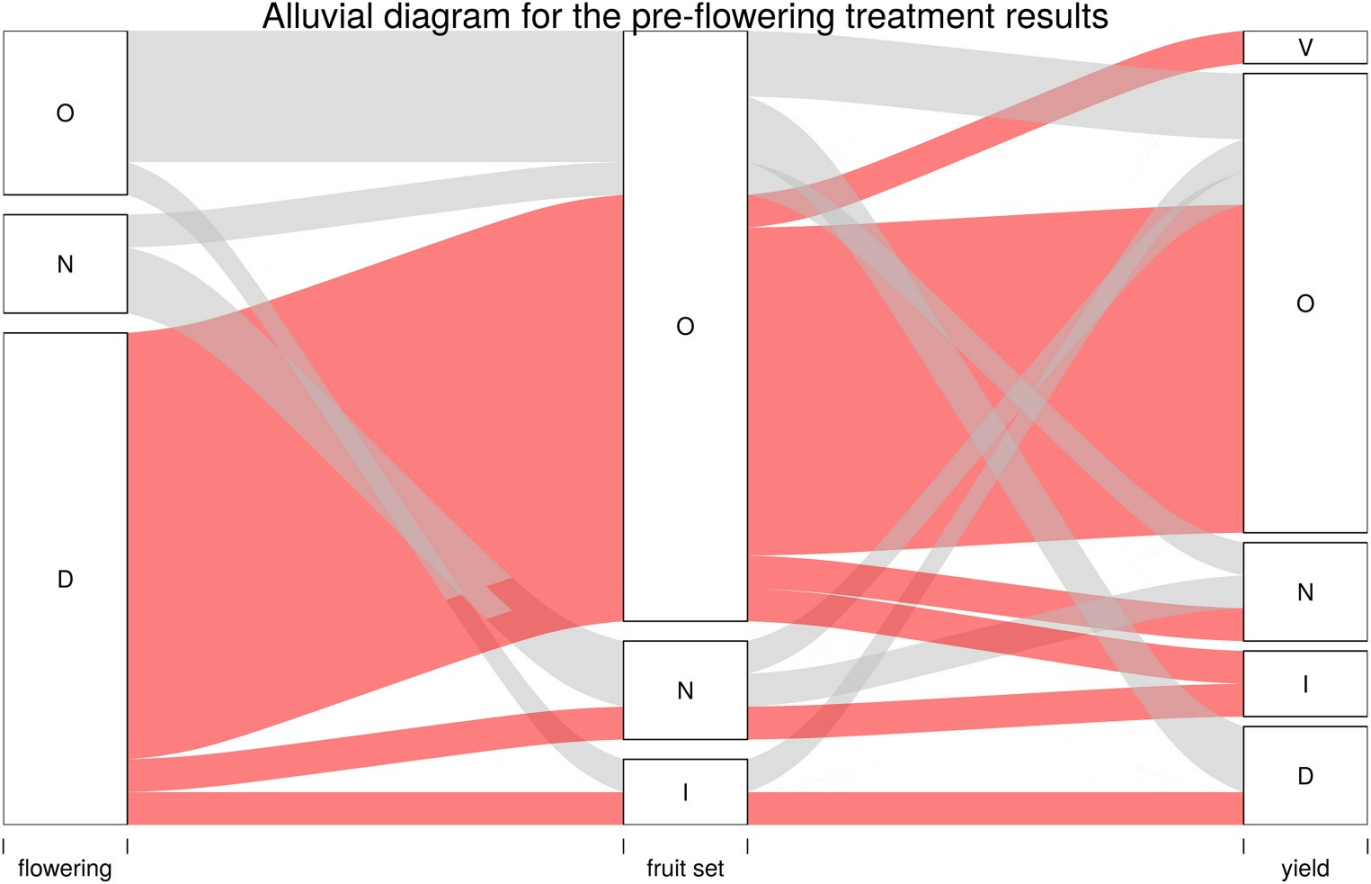
Alluvial diagram for the pre-flowering treatment results. Only the pre-flowering treatment studies were considered. The effect of GA pre-flowering treatments on flowering, fruit set and yield for each reviewed publication was noted as follows: N, no effect; I, increase; D, decrease; O, not studied and V, variable result. Bandwidth reflects the number of publications. The narrowest bandwidth represents a single publication. Red denotes the articles in which GA decreased flowering, while gray denotes the articles in which GA had a different effect.

**Fig 9:**
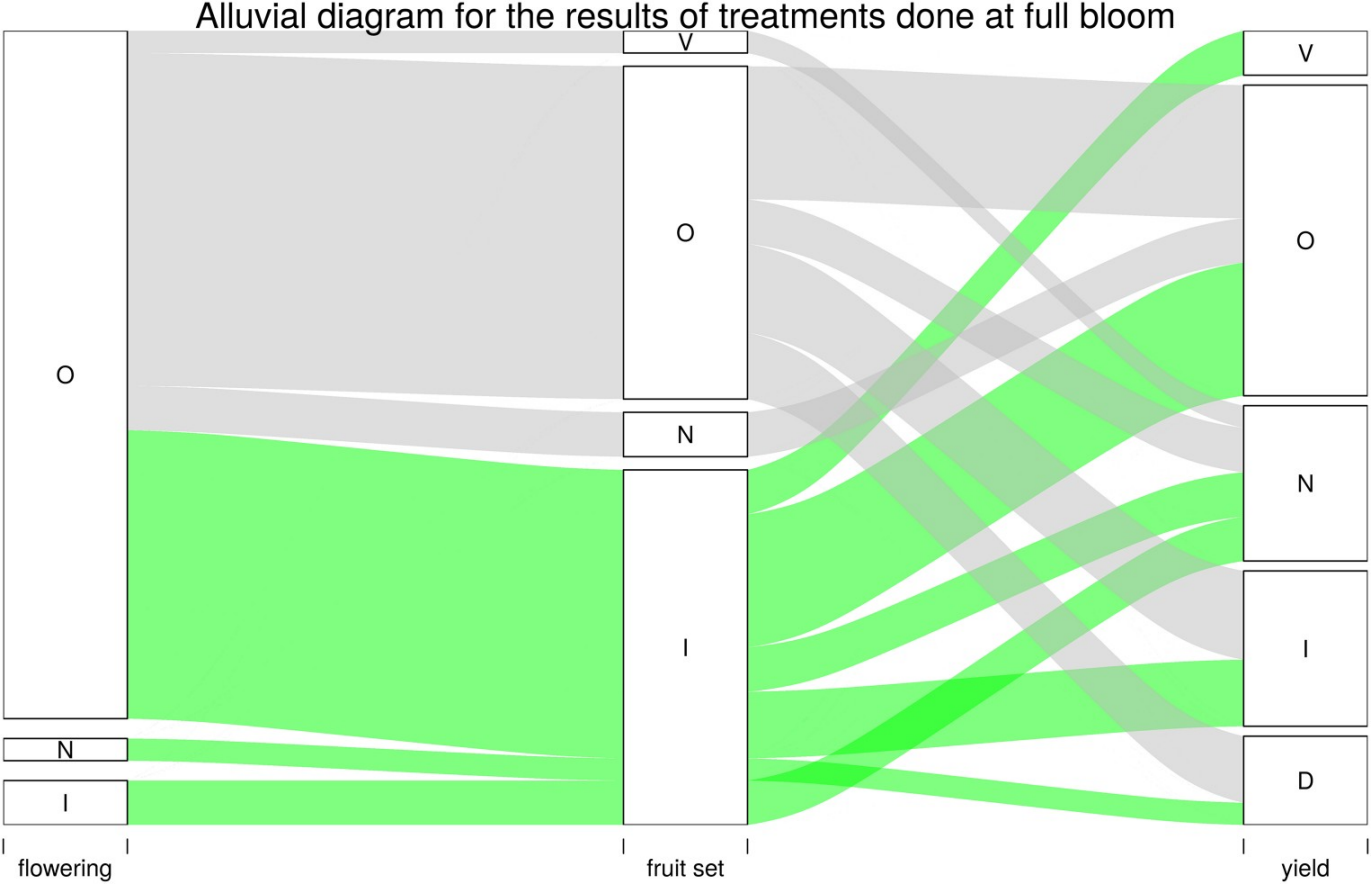
Alluvial diagram for the results of treatments done at full bloom or some weeks later. Only the studies with treatments at full bloom or some weeks later were considered. The effect of GA at full bloom on flowering, fruit set and yield for each reviewed publication was noted as follows: N, no effect; I, increase; D, decrease; O, not studied and V, variable results. Bandwidth reflects the number of publications. The narrowest bandwidth represents a single publication. Green denotes the articles in which GA increased fruit set, while gray denotes the articles in which GA had a different effect.

The results were charted according to whether their effect increased (I), decreased (D), had no effect (N), had a variable effect (V), or were not evaluated (O) upon flowering, fruit set and yield (Figs 8 and 9). Most publications with pre-flowering treatments in which the effect was evaluated (18 publications) agreed that GA decreases flowering (15 publications of 18), and only three observed no effect (S3 Table, subset pre-flowering and Fig 8). In most of these studies, the effect on fruit set and yield was not evaluated. When yield was evaluated, erratic results were obtained (Fig 8).

Studies with treatments at full bloom or some weeks later mostly reported increased fruit set (Fig 9), but these increases did not mean higher yields. The yield results were highly erratic as increases, decreases, no effect, or variable effects were found (Fig 9).

## Discussion

### Limitations for a critical evaluation of the results

Some limitations were identified with the reviewed reports, which make the results not easy to be evaluated or especially difficult to extrapolate to real field conditions. Most experiments were done on young trees (Fig 5C; 31% very young age 0–6 + 37.5% young age 6–12 years). Citrus growers often consider ‘adult tree’ to be a tree that reaches full production. Young and adult trees probably differed in terms of behavior, resilience and the relationship between vegetative and reproductive growth. For example, the ability to recover fruit set from stressful conditions during flowering would be much better in adult trees than in young trees [10,11].

One-year experiments were run in 79% of the reviewed studies (Fig 6). It is well-known that previous yield and tree ‘history’ affect current yields [9,72] and that first year treatments could affect second year production. Treatments like girdling can improve yield in the first year, but a negative long-term effect has already been pointed out [73,74]. Therefore, at least 3-year evaluations are desirable.

Most experiments used 3–6 trees per treatment, which means that researchers had as many as six yield data per treatment to analyze differences among groups. Yield, measured as the total yield per tree, is a crucial variable, and frequently shows a wide variability among individuals [48]. Therefore, a good amount of yield data is needed with a bigger experimental size.

The flowering and fruit set variables had bigger sizes (as they were measured on different branches), but sometimes used a confusing measurement methodology. Several authors suggested that significant differences were not correctly assessed because of the small sample sizes and the wide variability of some parameters used to quantify effects [75–77]. Spatial relationships are known to exist both between branches, based on their cropping history, and within branches in floral gradient terms [78]. Consequently, many reports fail to include crucial information about the dependent variables to make a critical evaluation of the results.

### GA basic mechanism in Citrus

If we analyze the effect of GA on the different citrus growth cycle phases, we find some common patterns.

It is well-known that GA induces organ elongation [1,79]. This growth response is the combined result of enhanced cell division activity and increased cell size [1]. Exogenous GA applications have clearly enhanced the growth of *Citrus* seedlings by increasing leaf number, stem length, internode length, root weight, and so on [2,3,40–43] (Table 3), therefore, stimulating cell division and vegetative growth.

The GA content of seedless Satsuma (*Citrus unshiu* [Mak] Marc. cv. Clausellina) and Clementine (*Citrus reticulata* [Hort.] Ex. Tanaka cv. Oroval) has been compared, with very interesting results [80,81]. Satsuma is a male-sterile cultivar that shows a high degree of natural parthenocarpy and good fruit set. Seedless Clementine varieties are self-incompatible, and display scarce fruit set ability in the absence of cross-pollination [81]. Upon petal fal the fruitlets of Satsuma and Clementine contained 65 and 13 picograms of GA, respectively. Endogenous GA levels have been consistently found to be higher in Satsuma than in Clementine [81]. Exogenous applications have improved set in Clementine, but have barely influenced Satsuma [81]. In addition, clementine fruits, in the absence of pollination, have presented an approximately 2-fold transient increase in free abscisic acid (ABA) content shortly after petal fall [80]. More evidence has revealed that net coverage reduces the amount of seedy fruit, but also the number of fruits and, consequently, the yield of Nadorcott (originated from cv. ‘Murcott’, *C*. *reticulata*). The adverse effect of net coverage on fruitlets drop could be eased by a single GA spray at full bloom [82].

All this evidence indicates the fact that seed formation (a process with a high cell division rate) is related with GA contents in young ovaries and is important for fruit set. A high degree of parthenocarpy varieties, like Satsuma, would have enough endogenous GA to set fruits without needing seed formation.

The source-sink relations and the role of GA have been analyzed in several studies [83,84]. Three treatments, emasculation, emasculation and self-pollination, and emasculation and GA have been used to study the translocation patterns of ^14^C-metabolites during flowering and fruiting in calamondin (*Citrus madurensis* Lour.) [83]. GA and self-pollination treatments have resulted in a considerably stronger mobilization of ^14^C-metabolites to young ovaries and developing fruits than when flowers were only emasculated and no further stimulus was provided [83]. In another experiment, BA (benzyladenine) and GA_3_ have enhanced ^14^C assimilate export by foliage to developing fruit, and GA_3_ has proven especially active in promoting fruit sink intensity (^14^C/dry wt) [84].

Countless evidence in citrus has shown that GA_3_ applications delay mature fruit senescence and fall [8,51–60]. In these cases, GA counteracts ABA (abscisic acid) action by delaying abscission. In some cases, a greener rind color has been associated with GA treatments [53,58] by activating rind growth and delaying global senescence. The data suggest that GA efficacy was not dependent on the application method, but on spray volume and GA dose [59].

Finally, the vast majority of experiments run on floral induction have shown that exogenous GA applications during the induction period reduce the quantity of flowers [85–90]. If we take into account that flowering is usually induced through stresses, such as low temperatures [91,92] or water stress [93], once again it would seem that GA has an antagonistic effect on stress hormones like ABA. In all cases, the induction response was proportional to the amount of stress [93]. Therefore, GA lowers the floral induction level, probably by counteracting stress hormones.

All this evidence points out a clear action of GA in citrus: GA is related to cell division and growth and, therefore, emphasizes the sink ability of the organ and discourages its abscission.

### GA effect on flowering and fruit set and why farmers do not always get the result they expect with GA applications?

Many of the reviewed studies applied GA to improve flowering, fruit set and yield with very variable results (Figs 8 and 9). For example, Koller et al. 1999 have shown that fruit production linearly decreased with increasing GA_3_, applied on July 22 and 29 (20 days before the spring bud), with no significant effect for GA_3_ applied upon the full bloom of ‘Monte Parnaso’ navel oranges [94]. Chao et al. 2006 have indicated that two GA_3_ treatments significantly reduced the kilograms and number of commercial fruit per Clementine mandarin tree in California [95]. Several studies have reported improvements in yield caused by GA treatments [82,96–100]. Yet why are these results so erratic?

Yield is the consequence of a long process that lasts from floral induction to harvest. During this process, many factors interact and GA levels are not always the main problem as many other factors can fail to limit yield.

Evidence can be grouped into: (i) treatments to control flowering (Fig 8); (ii) treatments upon full bloom, petal fall and several weeks later, to improve fruit set (Fig 9).

***Floral induction*** has been widely studied in citrus for a long time. Experiments under growth chamber conditions have shown that flower initiation can be induced by low temperatures (15°C/8°C) [91,92] or water stress [93]. The results have suggested that floral-promoting factors for induction (low temperatures, photoperiod, or water stress) can act alone or combined. In both cases (temperature and water stress), flowering response has been proportional to stress intensity (i.e., the more stress, the more flowering) [91–93]. Moreover in both cases, inflorescence type has been related with induction level: high induction level provokes mainly inflorescence with only flowers, and no or a few leaves, while low to moderate induction level provokes mainly inflorescences with more leaves than flowers [91–93]. Inflorescence type has been modified not only during the induction period, but also in initial flower shoot development phases [91,92]. Extreme water stress reduces flowering, and GA content became significantly higher in the leaves of trees under severe water stress than in the leaves of trees that endured moderate water stress [101,102]. Finally, it is well-known that fruits have an important inhibitory effect on flowering and that this effect depends on variety behavior [9]. This effect has been related to increased endogenous GAs due to fruit during the floral inductive period [14].

The main problems with flowering have been pointed out by Stover [10]. Light bloom can limit production, which usually happens in young trees that have not achieved full production, and also in “off” years of alternate bearing varieties [10]. However, heavy flowering can cause excessive crops, small fruit, and may increase alternate bearing severity [103]. Fruit size can be endangered by the early competition caused by heavy flowering, even when this heavy flowering is rapidly adjusted [104]. A high amount of carbohydrates and nutrients can be spent by organs (flowers and fruitlets) that will eventually fall; the availability of these materials for the following phases of growth may be impaired by this initial use [10,105]. In any case, a modest excess in reproductive efforts should not be passed over as it is helpful to adjust crop load through abscission of weak or damaged fruits [11], or as an insurance against climate adversities.

Therefore, light or heavy flowering can impair yield and consequently, control flowering, is an important tool for fruit tree farmers. However, one question remains: how can consistent moderate flowering be achieved under real field conditions?

In most of the reviewed studies (Fig 8), exogenous GA applications during the induction period consistently reduced flower formation [85,87,89,90,97,98,106,102,107–111], while the application of Paclobutrazol (PBZ), a known antagonist of GA, increased the number of flowers [90,108]. As when temperature and water stress were manipulated for floral induction, GA also affected inflorescence type. GA applications increased the amount of leafy inflorescences [107]. Indeed, it would seem that GA is able to counteract floral induction, probably by an antagonistic effect on stress hormones like ABA.

Most experiments were performed on young trees (Fig 5) under very controlled conditions and with repeated GA applications. GA applications were performed at any time during the induction period with good results, even on days before flowering [85,87,89,90,97,98,106,102,107–111]. It has been suggested that this technique can be used to mitigate alternate bearing in “on” years or to merely control the constant heavy flowering of some varieties [106,112]. The problem lies in the fact that floral induction load in adult trees and under real field conditions is very hard to estimate. This load depends on cold winter temperatures, rainfall, soil humidity, previous yield, and so on. Applying GA to counteract excessive floral inductions without exactly knowing the induction load is a blind step. The GA concentration and the number of applications to achieve the desired effect will depend on the previous induction load. If we reduce flowering too much, we can cause reduced yields. A predictive model with climate conditions, cultural practices, previous yield, etc., would be necessary to at least be able to classify (before flowering) the floral induction of the year as being very high, high, medium, low or very low and to, thus, make a decision about GA application.

Therefore, in this case, the mechanism and effect are clear and evidence coincides, but its correct use by farmers under field conditions still presents difficulties.

**Bloom and fruit set** are essential for yield. It has been already stated that highly natural parthenocarpy varieties, such as Satsuma, provide high endogenous GA content, while self-incompatible varieties, such as Clementine, present lower endogenous GA levels and some fruit set difficulties in the absence of cross-pollination [81]. Without pollination and a seed formation stimulus, Clementine ovaries have shown higher ABA levels and a clearly reduced yield [81,82]. It would seem that seed formation would increase GA endogenous levels, improve the GA/ABA ratio and, therefore, increase the sink ability of ovary and, thus, fruit set [83]. Accordantly, some citrus seedless varieties have fruit set difficulties and lower yields than cross-pollinated varieties.

Therefore, GA is a stimulus for fruit set that is naturally synthesized during seed formation. Consequently, the question is: why do exogenous GA applications not always improve fruit set and yield? Applications are effective when a reproductive stimulus is lacking, but not effective in other cases.

Talon et al 1992 have reported that GA_3_ applications at full bloom improve set in Clementine, but had barely any influence on Satsuma [81]. In general, GA applications have had no effect on fruit set when applied to Satsuma varieties [80,81,113]. Therefore, variety is essential for treatment effectiveness; some varieties do not respond to GA applications because endogenous levels are already high. When self-incompatible Afourer variety has been grown under nets, GA applications at bloom were useful for recovering yields up to control values [82]. Although more experiments are needed, it would seems that lack of pollination and a seed formation stimulus have been correctly replaced with GA applications.

GA has also been applied upon petal fall and some weeks later to improve fruit set with very variable results [13,83,96,98,108,114–118]. Many experiments have reported enhanced initial fruit set, but this effect was transient and the final yield did not increase in most cases [114,115,119,120]. Fruit set improvements does not necessarily mean yield improvements (Fig 9). These results indicate another limiting factor apart from GA. In these experiments, when GA applications have been combined with girdling or only girdling was done, production increased at least in the first year [98,114–117,119]. Girdling of branches increased the carbohydrate contents in leaves [117]. A few days after treatment, girdling increased the soluble sugars content in fruitlets, reduced the daily fruit drop, and diminished abscission [121]. Most evidence suggests that carbohydrate availability limits yield more than GA levels [119]. According to the reviewed studies, girdling is the most powerful technique to improve fruit set and yield, probably by modifying carbohydrate distribution, but with uncertain consequences for the next years [116,117,122]. Erner (1988) pointed out that girdling is a risky procedure. Annual girdling can damage severely trees by restricting root growth and leading to excess flower bud differentiation [74].

Hofman 1990 have compared leafy and leafless inflorescences and obtained very interesting results [123]. The mass of the fruitlets adjacent to young leaves (“leafy” fruitlets) was bigger than the fruitlets adjacent to mature leaves (“leafless” fruitlets); leafy fruitlets displayed considerably greater tree retention after 10 weeks (12.7%) than leafless fruitlets (1.2%) [123]. The GA and ABA concentrations between the leafy and leafless fruitlets were compared, but with no significant differences. Once again, this means that the carbohydrate availability supplied by leaves is crucial. Moreover, Mehouachi et al. (2000) have reported that defoliation shortly after anthesis increases fruitlet abscission (up to 60%) over the following 12 weeks. Defoliation does not substantially modify endogenous GA levels, but increases ABA contents [124,125].

Some authors think that GAs are the main determining factors for early fruit set, while the subsequent growth of developing fruits depends mostly on carbohydrate availability [126,127].

When fruit-set increased excessively by GA applications in many experiments, a clear reduction in the average fruit weight was recorded, which means lower commercial values [114,117,128].

The sink ability of leaf shoots can also be modified by GA applications which can, therefore, change the relationship between vegetative and reproductive growth [129–131]. Indeed two opposite effects have been highlighted: (i) a positive one: the new young leaf generates carbohydrates to nourish fruitlets; (ii) a negative one: the initial carbohydrates demanded by vegetative shoots can impair fruit set [129–131].

Finally, effects of GA applications have been compared between “on” and “off” years. GA_3_ increased the yield of commercially valuable ‘Nules’ Clementine fruit, but only in the off-crop year of an alternate bearing orchard. In the following on-crop year, it was better not to apply GA_3_ because no GA treatment increased either total yield or fruit size [72].

Therefore, if yield problems are caused by lack of a reproductive stimulus for fruit set, GA applications will be effective. However, lack of stimulus is not always the problem. Production can be harmed by improper floral induction, carbohydrate availability, inflorescence types, climate conditions in critical phases like summer fruit drop, and so on. In all these cases, GA applications will have no effect on yield.

Minor sources of variation have been pointed out, like the solution pH [132] or environmental factors [23]. Guardiola et al. 1988 have reported that the effect of growth-regulators on fruit growth is markedly affected by small differences in application dates [24]. It has been suggested that the maximum effect of GA applications on flowers coincides with endogenous contents of gibberellins and cytokines upon flowering opening [113].

## Conclusions

Most publications with pre flowering treatments agreed that GA decreases flowering, while only 3 out of 18 did not observe any effect. Studies with treatments at full bloom mostly reported increased fruit set. However, these increases did not imply higher yields. The results on yield were highly erratic. After this systematic review, many reports support these results.

The main limitations of the experiments done to evaluate the action of GA applications on flowering, fruit set and yield were tree age, number of evaluated years, few data for total yields, and sometimes a confusing flowering and fruit set evaluations. Bigger experiments lasting at least 3 years on rows of adult trees would be desirable to acquire more yield data.

For floral induction, the GA effect has been widely proven. GA applications counteract the floral induction caused by stress by reducing flowering. However, on adult trees under field conditions, this technique would be difficult for farmer to apply because it would be necessary to previously estimate the natural floral induction of trees. This natural induction is controlled mainly by climate factors and previous yield.

For fruit set, results showed that AG applications can improve fruit set, but not necessarily improve yield. During flowering and fruit set, many problems can arise that limit production. Some examples are: excessive number of leafless inflorescences, excessive early competition caused by heavy flowering, low carbohydrate availability sometimes caused by depletion due to previous yields, or stressful climate conditions in critical fruitlet drop phases. Only when the problem is lack of stimulus for fruit set will GA applications improve yield.

Considerable evidence suggests that, in most cases, the main factor to limit yield would be carbohydrate availability rather than GA levels. In other words, tree holding capacity or the ability to nourish fruitlets. Perhaps the easiest way to improve yield is to improve the health, strength and balance of citrus trees throughout the year.

If a tree is forced over its capacity with GA applications, small fruits (with a low commercial value) and depleted reserves for the next year will be obtained. Yet when a tree produces beneath its capacity and this reduction is caused by lack of stimulus (e.g. when growing under nets or in “off” years), GA applications will be effective in improving yield. If yield is low because tree capacity is low (depleted by previous yields or unhealthy trees), or because excessive early competition is caused by heavy flowering, GA applications will not improve yield. Each variety will display a different behavior and will, therefore, need a different solution.

## Supporting information

S1 Table Group definition and content. (XLSX)

S2 Table Initial data collection. (XLSX)

S3 Table Database extracted from the sub-set of publications. (XLSX)

S1 PRISMA Checklist Items checklist grouped in section/topic of PRISMA statement. (DOC)
